# Fast Hydrogen Detection via Optical Fibers Coated with Metal Hydride Thin Films

**DOI:** 10.3390/s26113285

**Published:** 2026-05-22

**Authors:** André D. Santos, Miguel A. S. Almeida, João P. Mendes, José M. M. M. de Almeida, Luís C. C. Coelho

**Affiliations:** 1 INESC TEC—Institute for Systems and Computer Engineering Technology and Science, Rua Dr. Roberto Frias, 4200-465 Porto, Portugal; andre.d.santos@inesctec.pt (A.D.S.); miguel.a.almeida@inesctec.pt (M.A.S.A.); joao.p.mendes@inesctec.pt (J.P.M.); luis.c.coelho@inesctec.pt (L.C.C.C.); 2 Department of Physics and Astronomy, Faculty of Sciences, University of Porto, Rua do Campo Alegre s/n, 4169-007 Porto, Portugal; 3 Department of Engineering Physics, Faculty of Engineering, University of Porto, Rua Dr. Roberto Frias, 4200-465 Porto, Portugal; 4 School of Science and Technology, University of Trás-os-Montes e Alto Douro, Quinta de Prados, 5000-801 Vila Real, Portugal

**Keywords:** hydrogen sensor, optical fiber sensors, metal hydrides, magnesium, thin films

## Abstract

Detection of leaks in hydrogen (H_2_) infrastructure is required on a large scale to enable a safe widespread use of this clean energy source. Sensing solutions must be low-cost, use scalable fabrication methods and allow multiplexed detection while providing reliable safety alarms as fast as possible. Optical methods can make this possible while avoiding the risk of ignition due to electronics at the point of detection. Metal hydride-based micro-mirror configurations benefit from a simple interrogation scheme, as long as the sensitive element can produce a large optical response. Magnesium thin films undergo a drastic variation of properties when hydrogenated, making them suitable for this application. In this work, a micro-mirror device using single-mode fibers capable of detecting the presence of H_2_ with a loading t_10_ and t_90_ of 1.2 and 3.0 s, respectively, is demonstrated. A complete interrogation unit was developed, presenting a solution suited for widespread deployment using industry-standard optical components and equipment.

## 1. Introduction

Hydrogen (H_2_) is, currently, a significant part of many initiatives for the transition to cleaner energy vectors and sources. Just recently, in the context of COP29, in Azerbaijan, 62 countries pledged to speed up the production of low-carbon hydrogen and include it in their national energy and climate programs, through the “COP29 Hydrogen Declaration” [1]. This is happening in recognition of several advantageous characteristics of H_2_—namely, its unparalleled energy density and the lack of environmentally harmful combustion byproducts [2]. A key point of the above-mentioned initiatives is the widespread construction and repurposing of gas infrastructure for H_2_, with tens of thousands of kilometers of pipelines planned to enter operation by 2030 [3]. Together with other structures such as refueling posts or storage tanks, these result in a growing demand for solutions that can ensure safety and maintain public trust.

Due to hydrogen’s wide flammability range (4–75 vol.%) and low ignition energy, this will require adequate monitoring of concentration and quick detection of leaks at any number of points [4]. For leaks in particular, signaling the presence of H_2_ as fast as possible to prevent fires and explosions is a priority, rather than determining an exact concentration. There is a wide variety of available sensing methods—resistive, electrochemical, catalytic, etc.—but optical sensors have emerged as advantageous for these applications in large part due to the possibility of avoiding the presence of electronics at the point of detection, eliminating the risk of ignition due to sparks or discharges [5]. The use of optical fibers and industry-standard technologies, specifically single-mode fibers (SMFs) and C-band wavelengths (1530–1565 nm), makes other beneficial features such as remote detection or immunity to EM interference possible, while lowering overall costs. Methods employing fibers show a great variety, including intensity measurement, the use of gratings (FBGs), those based on the surface plasmon resonance phenomenon (SPR) [6] or interferometric techniques. Simple configurations like the “micro-mirror” approach can be easily fabricated using physical vapor deposition techniques, requiring little more than guiding optical power to a structure on the tip of a fiber and measuring the reflected intensity.

The use of metal hydrides as micro-mirrors in optical fiber hydrogen sensors has already been shown, for example, by Butler [7], Slaman et al. [8], Yi et al. [9] or more recently, by Verhoeff et al. [10]. In particular, Slaman et al. present a sensitive structure that includes thin films of magnesium (Mg) alloys, such as Mg-Ti or Mg-Ni. Magnesium is considered to have the most drastic variation in optical properties when undergoing its phase transition to a hydride state, compared to other metal hydrides [11], thus providing a larger response amplitude in sensors with intensity measurements, which usually lack the sensitivity of more complex interrogation methods.

Direct hydrogenation of a Mg film does not, however, occur at ambient temperature and pressure. The use of a thin palladium (Pd) catalyst layer above the film can promote dissociation and adsorption of H_2_ molecules while also protecting from oxidation [12]. There is also the issue of reversibility: magnesium hydride (MgH_2_) is highly stable, and efficient dehydrogenation usually requires high pressures and/or temperatures well above ambient conditions [13].

While the use of the aforementioned alloys is known to offer improved desorption kinetics and reversibility, the fabrication of these alloys with specific optimal compositions may present financial or technical hurdles to the large-scale implementation or adoption of Mg-based sensors. Meanwhile, the use of simple Pd-capped Mg films has not been investigated enough regarding their applicability in fast sensors for the presence of H_2_. Furthermore, the measurement of very small response times (on the order of a few seconds) specifically for micro-mirror H_2_ sensors that use these materials is rarely reported in the literature. Apart from a few examples, such as Yi et al. with a 6 s loading time, Slaman et al. with 12.5 s, or Liu et al. [14] with a t_90_ of 200s at 4 vol.%, these metrics are usually omitted or not measured due to the lack of an appropriate setup (as mentioned by Verhoeff et al., for example). Even when a faster response is obtained, the amplitude of signal variation may be small, making detection more difficult and increasing interrogation equipment costs.

In this work, we demonstrate the feasibility of quick H_2_ detection with Pd-capped Mg thin films, and show how such micro-mirror devices can be fabricated on SMFs using scalable methods, obtaining structures capable of signaling the presence of H_2_ extremely quickly, with an easily detectable variation in the optical signal. Additionally, a standalone interrogation unit prototype is presented, providing a proof-of-concept for a remote and multi-point leak detection system.

## 2. Materials and Methods

The optical behavior of thin film structures consisting of Pd-capped Mg layers (on glass substrates) was simulated using the Transfer Matrix Method (TMM), with the variation in reflectance between the metallic and the hydride phase being calculated using refractive index data from Palm et al. [11]. This result was used to determine an optimal range of layer sizes for Mg and Pd, maximizing optical contrast while maintaining an overall low thickness, for quickly reaching a higher degree of hydrogenation in the structure.

### 2.1. Fabrication

Initially, the hydrogenation at ambient conditions of Pd-capped Mg films, of thicknesses around 100 nm, was verified using planar glass substrates. The samples were fabricated via RF Magnetron Sputtering, using a custom-built setup which allowed for the deposition of protective polymer layers. The use of a multi-target system proved necessary due to the quick oxidation of the Mg film between vacuum cycles, blocking hydrogen diffusion. Then, 20 nm Mg films capped with 10 nm of Pd were deposited on the tips of Corning SMF-28 single-mode fibers. This was repeated with the addition of a 30 nm polytetrafluoroethylene (PTFE) protective layer to investigate its impact on the sensor’s performance. In all instances, deposition of Mg, Pd, and PTFE was carried out with applied RF powers of 12 W, 12 W, and 20 W, respectively, with film thickness being monitored using a high-resolution quartz microbalance.

### 2.2. Characterization

**Figure 1:**
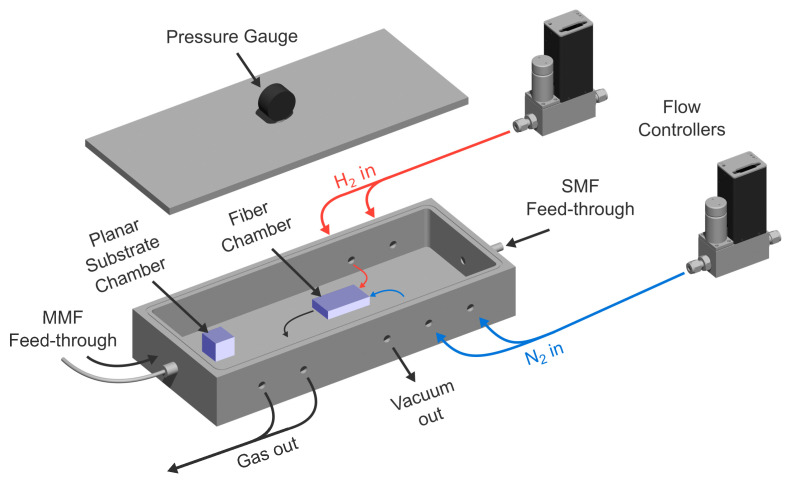
Diagram of the controlled gas flow setup used for characterization of the produced planar and fiber samples.

All samples were characterized at room temperature and ambient pressure in a controlled gas flow setup (Figure 1), which includes a sealed chamber with gas inlets/outlets, optical fiber feed-throughs, and a pressure gauge. Gas flows are routed into the chamber using mass flow controllers, which are managed via computer with custom-built software developed for this purpose.

**Figure 2:**
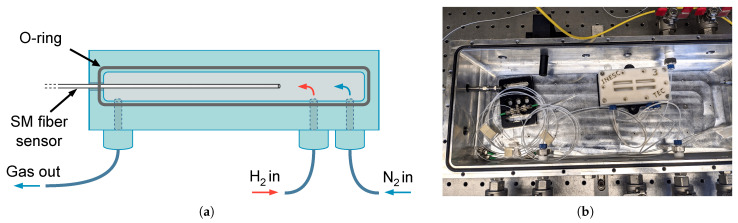
(**a**) Diagram of the inner chamber used for fiber samples (**b**) Photograph of the same inner chamber placed inside the outer gas chamber.

For planar substrates, substrate-side reflectance spectra were monitored by inserting samples into a smaller inner chamber, placed inside the outer chamber. This allowed light coming from a multi-mode (MM) fiber feed-through to hit the substrate and be reflected back, while gas was made to flow across the sample’s top side by connecting outer chamber inlets to holes in the inner chamber’s top piece. An O-ring, tightly pressed against the sample, avoided leakage of this flow into the surroundings. The sample’s top side was exposed to a 4 vol.% H_2_ flow after a few minutes of a 100% N_2_ flow. As for the fiber samples, a different setup was used (Figure 2). The fiber tips were inserted into a different inner chamber along which the gas is made to flow, covered with a glass slide which is pressed against an O-ring with a screwed-on lid. The inner chambers were placed inside the large sealed outer chamber depicted in Figure 1 for safety reasons, preventing leakage of test gases into the surrounding environment.

In order for measurements of very low response times to be possible, H_2_ and N_2_ flows of 300 mL/min are routed separately and fed directly to the fiber tip, minimizing the delay between the change in flow at the mass flow controllers and the actual exposure of the sensing structure to hydrogen. After accounting for the volume of tubing and the error introduced from electronic noise, an upper bound of 300 ms was calculated for the error in the determination of the response times. IR reflectance spectra were recorded using an optical spectrum analyzer, while all time-domain reflectance measurements for fiber samples were made using the interrogation system specifically developed for this work, which employs a 1550 nm fiber-coupled laser diode as a source.

### 2.3. Interrogation System

**Figure 3:**
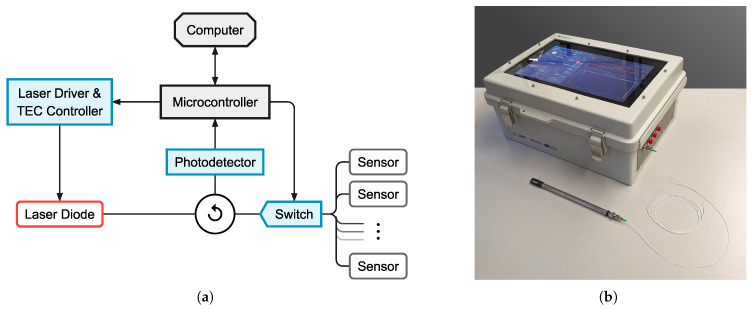
(**a**) Block diagram of the developed interrogation system. Simple lines denote optical fiber connections, while lines with arrowheads denote electrical connections. (**b**) Photograph showing the prototype unit, with one detection probe connected. The screen shows the developed user interface.

A working prototype for a standalone interrogation system was developed, encompassing a 1550 nm laser diode (XYT Sharetop CWDM TEC Pigtail Laser Diode) with built-in thermoelectric temperature control, a laser driver (Wavelength Electronics LDTC0520) capable of setting the diode’s current and temperature, an InGaAs PIN photodiode (EPM 606LL) and a MEMS optical switch (Optosun 1 × 4 Mini Size). A microcontroller unit controls these devices and acquires the photodetector’s signal, communicating with a computer. Finally, a circulator distributes the optical power from the laser diode to the optical switch, being reflected at one of the several detector fiber tips that can be connected and routed back to the photodetector. This architecture is shown in Figure 3a.

The computer, embedded into the prototype, can acquire data and monitor the system at a rate of up to 25 Hz. A custom-built software application was specifically developed to control the laser output and channel switching, while providing real-time visualization and the possibility of saving data. It is also able to detect quick decreases in the incoming signals and show alarm warnings based on configurable thresholds.

An enclosure for the sensitive fiber tips was designed, consisting of a hollow steel tube with evenly distributed slits on one of its ends. A similarly slitted cap covers this area, with a sheet of micro-porous mesh underneath. The mesh, with a hole size of 15 μm, protects the fiber from dust, mechanical and chemical damage, without restricting airflow. On the opposite end, a connectorized SMF pigtail is spliced to the fiber, providing an easy connection method. Figure 3b shows the prototype, with a detection probe, connected to one of the outputs.

## 3. Results

### 3.1. Pd-Capped Mg Films

TMM calculations showed, for a Pd-capped Mg thin film, that while the reflectance variation upon hydrogenation predictably tends to increase for higher film thicknesses, there is a local maximal region for lower thicknesses, spanning ranges of 20 to 30 nm for Mg, and up to 10 nm for Pd. This is desirable because shorter diffusion paths lead to faster optical responses for the sensitive structure [15]. The calculations are available in the Supplementary Information.

**Figure 4:**
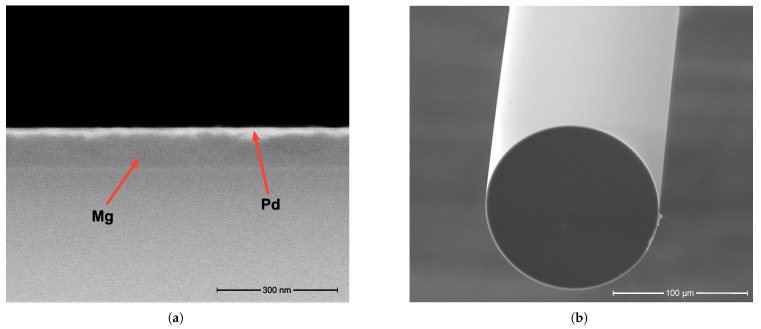
SEM images of: (**a**) a planar substrate with a thin film stack of Mg (100 nm) and Pd (10 nm); (**b**) the tip of an SMF, where a Pd-capped (10 nm) Mg film (20 nm) was deposited. The magnification used was (**a**) 300,000× and (**b**) 1000×, with an accelerating voltage of 15 kV in both images.

The deposited stacks were validated using scanning electron microscopy (SEM) on a FEI QUANTA 400 microscope, where the different layers of a Mg-Pd structure were observable on planar substrates (Figure 4a). Energy-dispersive X-ray spectroscopy (EDS) measurements were also used to validate their composition (see Supplementary Information). Samples produced while venting the sputtering chamber between each layer (to switch targets) showed only a very small response to 4 vol.% hydrogen, attributable to the hydrogenation of the Pd cap layer. Meanwhile, those fabricated in a single vacuum cycle, using a multi-target system, had a markedly greater response, measured as a sharp drop of over 30% in reflectance. Their appearance was also visibly changed, from highly reflective to a dark brown, more transparent region where the film was exposed to the gas.

The addition of a protective PTFE layer of 30 nm showed no negative impact on the sensing performance, in accordance with what is commonly reported [15,16]. Only a slight overall reflectance offset of about 2% is apparent, presumably an increase in attenuation due to the presence of the polymeric layer.

### 3.2. SM Fiber Detectors

**Figure 5:**
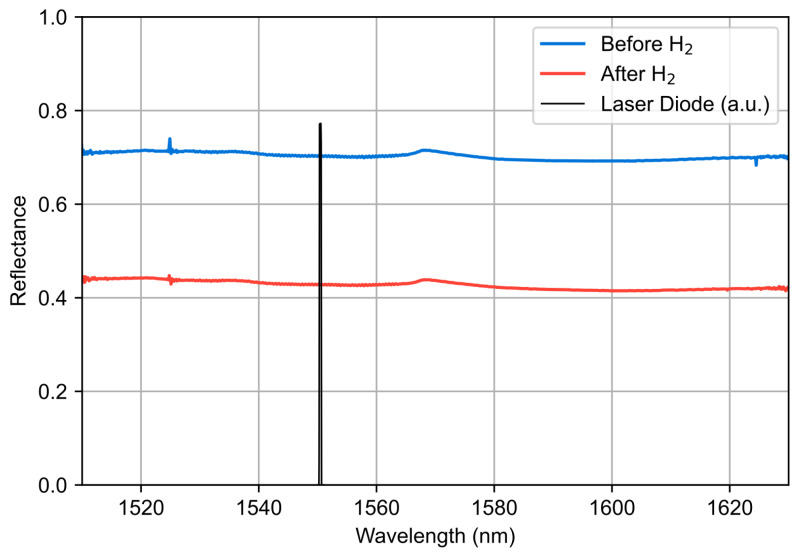
IR reflectance spectra of the optical fiber detectors, before and after exposure to H_2_. The black line indicates the laser diode output spectrum used for sensor interrogation (1550 nm).

Figure 4b shows the tip of one of the sensors produced on SMFs, where a homogeneous coating of the tip is visible. The Mg-Pd-PTFE structures produced significant and very fast responses to exposure to a 4 vol.% H_2_ flow. Infrared reflectance spectra of these sensors, presented in Figure 5, show a marked decrease upon exposure, in agreement with TMM calculations.

**Figure 6:**
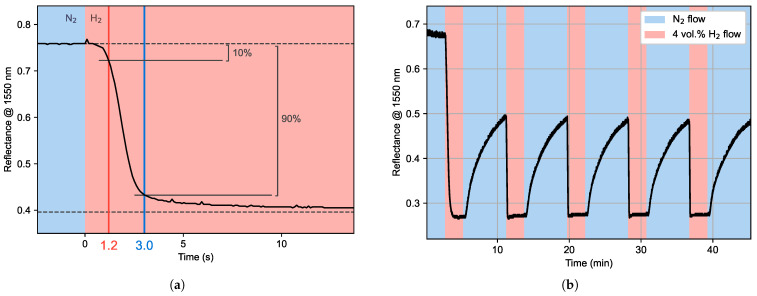
(**a**) Measured reflectance at 1550 nm for a fabricated Mg-Pd detector upon first exposure to a 4 vol.% H_2_ flow. Red and blue vertical lines indicate the loading t_10_ and t_90_, respectively. (**b**) Measured reflectance at 1550 nm for a fabricated Mg-Pd detector for five loading–unloading cycles.

The sensor response was also characterized in the time domain, using the interrogation system mentioned above. Figure 6a shows one such instance, where the reflectance decreased by 36%, with the initial 10% of this variation occurring within 1.2 s of the change in gas flow content. The majority of this decrease (90%) is attained within 3.0 s. Because the signal variation is so large, the alarm for detection of a leak can be triggered before the full variation occurs, since even a fraction of it is easily discernible. In this context, the definition of t_10_, commonly employed to describe the recovery time, can also be applied to describe the initial dynamics of the detector’s loading response, giving a better sense of the time required for detection in a real application scenario. On the other hand, a fast recovery is not paramount for a sensing device that should only be triggered infrequently.

The performance of the detectors was also evaluated for repeated exposures to hydrogen, by applying a 4 vol.% H_2_ flow for 150 s followed by a 100% N_2_ flow for 360 s. As seen in Figure 6b, although unloading is considerably slower than loading, reversibility is achieved and several consecutive detections are possible.

It should be noted that, as is clear from Figure 6b, the original level of reflectance is not recovered during these cycles. This is because waiting the required time for complete desorption of H_2_ from the detectors was not feasible, as a full recovery can be very slow. Thus, rather than the recovery time, the focus was verifying reversibility to make repeated detections possible, while studying the response time in more detail.

**Figure 7:**
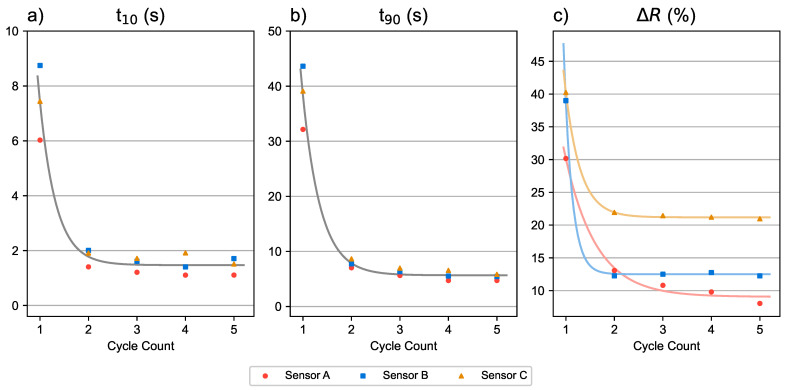
Performance of three fabricated detectors for the first five loading–unloading cycles at 4 vol.% H_2_: (**a**,**b**) loading response t_10_ and t_90_; (**c**) reflectance variation upon H_2_ exposure.

Figure 7 shows the cycling response in several detectors from the same fabrication batch. It should be noted that these are not the same detectors as the ones presented in Figure 6, and had not been previously exposed to H_2_. The loading t_10_ and t_90_ drop sharply across all detectors from cycle 2 onwards, and their performance becomes highly homogeneous. However, the same is not true regarding their reflectance variations (ΔR), which appear to settle at different values. While some of this behavior can be attributed to the lack of complete desorption during the applied cycles, for the reasons explained above, a “pre-cycling” step before deployment could help ensure a consistent and fast performance.

### 3.3. Response Time vs. Concentration

The impact of H_2_ concentration on the detector response time was also studied. Several detectors were subjected to sets of five cycles (similarly to what is shown in Figure 6b) using different concentrations from 0.5% to 4%. These were obtained by diluting the flow from 4% and 1% H_2_ sources with appropriate N_2_ flows, with 0.5% being the lowest concentration that could reliably be achieved with the current setup. The detectors promptly indicated the presence of H_2_ at the various concentration levels.

**Figure 8:**
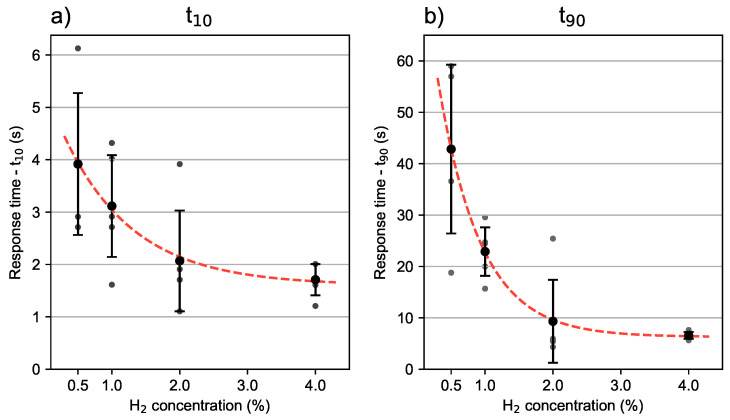
Average loading performance of fabricated detectors for their 5th cycle loading depending on the concentration of H_2_ used. (**a**) The 5th cycle loading t_10_. (**b**) The 5th cycle loading t_90_. Red dashed lines indicate trends.

Figure 8 shows the mean loading t_10_ and t_90_ for the 5th cycle, with clear trends indicating that the response time is lower for higher concentrations. This is more noticeable for the t_90_, while a few-second t_10_ is still achieved down to a 0.5% concentration. This suggests a wide range of operation for detection, starting at 0.5% H_2_.

### 3.4. Cross-Sensitivity

Several molecules are consistently identified in the literature as possible interferents for Pd-based hydrogen sensors. Among these, methane (CH_4_), ammonia (NH_3_), carbon dioxide (CO_2_), carbon monoxide (CO) and water vapor are considered the most relevant [17]. CO_2_ and CO can be adsorbed by Pd and contribute to the fouling of this layer through the formation of palladium oxide (PdO), but this effect is not the most impactful when taking into account the amounts commonly found in the atmosphere. Meanwhile, water vapor is highlighted as the biggest contributor to this process [18]. Thus, PTFE is highly favored as a protective material due to its hydrophobic properties [19].

**Figure 9:**
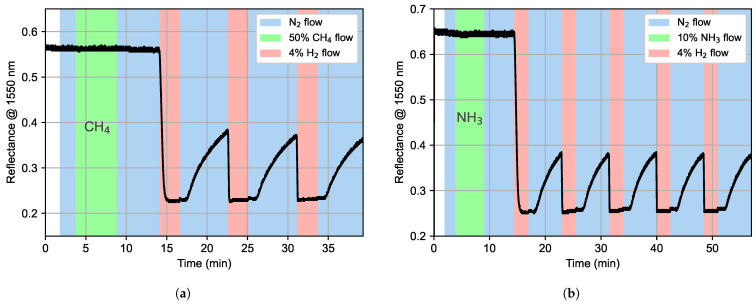
Detector response in cross-sensitivity tests to CH_4_ (**a**) and NH_3_ (**b**), showing the absence of reflectance variation when exposed to the interferent gas.

As shown in Figure 9, cross-sensitivity with CH_4_ and NH_3_ was tested by exposing the detectors to each of these interferents for a period of 5 min, before applying several loading–unloading cycles, verifying their correct performance. In both cases, there was no response to the interferent, and no discernible alteration in subsequent detection of H_2_.

## 4. Conclusions

As shown in this work, Pd-capped Mg thin films can provide a very fast yet ample optical variation that can enable near-second detection of the presence of H_2_. While it is not feasible to extract information about the environment’s hydrogen concentration using these materials and techniques, this method nevertheless provides a solution to the demand for faster H_2_ monitoring. This is particularly relevant in contexts where one could expect the absence of hydrogen during normal, correct operation, as is the case for leaks in infrastructure. Additionally, interrogation is not restricted to particular wavelength ranges, meaning that single-mode fibers and standard C-band wavelengths can be used, making implementation easier and less expensive, while enabling remote detection.

This type of device is, however, inherently ill-suited for repeated detection, due to poor unloading. For situations where exposure to hydrogen should be infrequent, it allows the use of a simple intensity-based interrogation technique, while the fabrication process itself is potentially quite cost-effective and highly scalable. Thus, the downside of requiring a replacement of sensing probes following the hopefully rare event of infrastructure malfunction may be offset by the ease and lower-cost of manufacturing them. For the purposes of demonstrating the sensor’s functionality, and with field testing in the near-future in mind, an interrogation system was developed and used in the characterization of all fiber-based samples in this work, showing a path for the application of this technology. It is able to monitor several detectors simultaneously, making multi-point detection possible for coverage of large infrastructure. In future work, the thicknesses of the sensitive layers could be further optimized, mainly by reducing the Pd layer while still ensuring an homogeneous film.

## Data Availability

The original contributions presented in this study are included in the article material. Further inquiries can be directed to the corresponding author.

## Supplementary Materials

The following supporting information can be downloaded at: https://www.mdpi.com/article/10.3390/s26113285/s1, Figure S1: Comparison of variation in reflectance spectra upon full hydrogenation for Mg and Pd thin films with a thickness of 30 nm, atop a glass substrate; Figure S2: Comparison of variation in reflectance spectra upon full hydrogenation for a Mg thin film of 30 nm atop a glass substrate, with (b) and without (a) a Pd capping layer of 10 nm; Figure S3: Calculated variation of reflectance upon hydrogenation at 1550 nm for a multi-layer structure with variable thicknesses of Mg and Pd. White contour lines indicate total structure thickness, while the red dashed line indicates boundaries of a region with high reflectance variation and low total thickness; Figure S4: EDS Spectrum for a 20 nm Mg film capped with 10 nm Pd, on a silicon substrate; Figure S5: Diagram of the sensor, showing an exploded view of the optical fiber and its enclosure. The insets show an assembled view (top right) and a shematic of the optical fiber tip with the deposited thin films (bottom left); and Figure S6: Photograph showing several sensor parts in different stages of enclosure assembly.

## Abbreviations

The following abbreviations are used in this manuscript:

SMF	Single-mode Fiber
MMF	Multi-mode Fiber
FBG	Fiber Bragg Grating
SPR	Surface Plasmon Resonance
TMM	Transfer Matrix Method
PTFE	Polytetrafluoroethylene
SEM	Scanning Electron Microscopy
