# Supplementary material for: Fast Hydrogen Detection via Optical Fibers Coated with Metal Hydride Thin Films
Source: Sensors (Basel). 2026 May 22;26(11):3285. doi: 10.3390/s26113285 (PMC13258869; doi:10.3390/s26113285)
Supplement: Supplementary file 1 [file sensors-26-03285-s001.zip › sensors-4257593-supplementary.pdf]

Transfer Matrix Calculations

The following section details the calculations of optical behavior of metal hydride thin films, whose results were used to inform the remainder of the work presented in the manuscript. The calculations used the Transfer Matrix Method, which was implemented in Python using modules such as NumPy for fast vectorized operations following the matrix formalism as outlined by Saleh and Teich (*Fundamentals of Photonics, 2nd edition*. in Wiley Series in Pure and Applied Optics. John Wiley & Sons, 2013).

As a starting point, Figure S1 shows calculated reflectance spectra (covering visible and infrared (IR) ranges) for 30 nm films of Mg and Pd. Their metallic phases are compared with the respective hydrides. It is apparent that the optical properties change significantly for both in a similar way. As expected, Mg shows the largest variation in reflectance across the entire spectrum (over 55%). Both Mg and Pd films have significantly lower reflectance in their hydride phases.

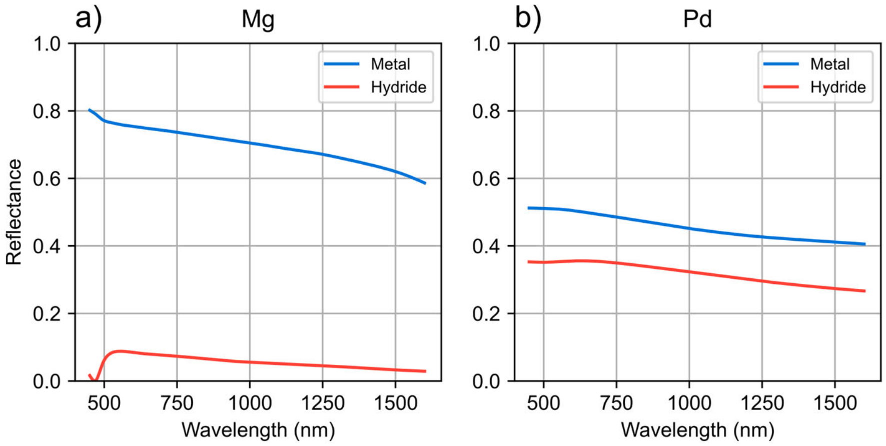

Figure S1. Comparison of variation in reflectance spectra upon full hydrogenation for **(a)** Mg and **(b)** Pd thin films with a thickness of 30 nm, atop a glass substrate.

Figure S2 shows how the addition of a 10 nm Pd cap layer to a 30 nm thin film impacts the reflectance of the whole structure. There is a consistent optical behavior across most of the presented spectral range, particularly in the SM fiber low-loss region (1200 to 1650 nm). The "flatness" of these spectra means that, in theory, the optical response of these structures when exposed to H_2 should not vary significantly with the wavelength used for interrogation. While operation in the IR range is preferred, this is an advantage in terms of ease of implementation of this sensing method. It also means that results obtained at different wavelengths can still be useful and, in some sense, compared.

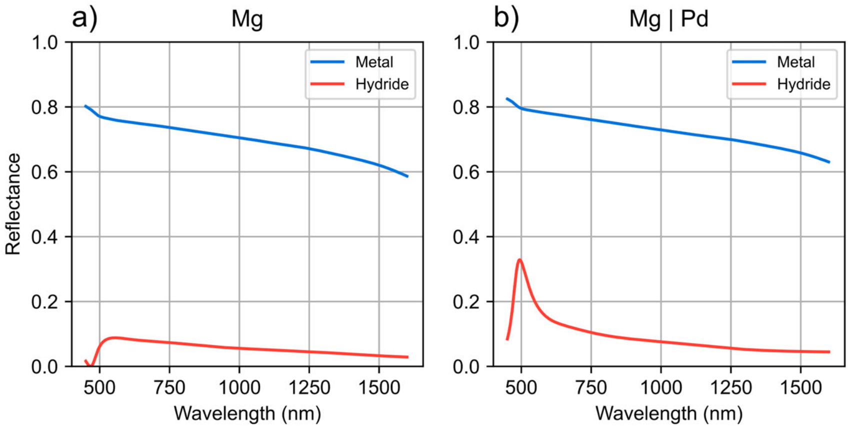

Figure S2. Comparison of variation in reflectance spectra upon full hydrogenation for a Mg thin film of 30 nm atop a glass substrate, with (**b**) and without (**a**) a Pd capping layer of 10 nm.

TMM calculations were also used to predict ranges of film thicknesses that optimize the sensing structure's response. To do this reflectance of the whole structure was calculated, from the substrate, at a wavelength of 1550 nm, while layer thicknesses were left as parameters. The value of interest is the difference between this result in the metallic and the hydride phases.

Figure S3 shows a 2D colormap of the reflectance variation at 1550 nm between the two phases. The "x" and "y" axes represent a variation in the Mg and Pd film thicknesses, respectively. The total structure thickness, which is just the sum of that of both films, is contour-mapped with white lines. The behavior shown in the plot follows some intuitive trends. The reflectance variation goes to zero at the origin, since it is the absence of film material. Meanwhile, at very low Mg thicknesses, only very low variations are observed, even for larger Pd films. This is consistent with the optical properties of each metal hydride discussed above. Finally, the structure's reflectance variation becomes much higher for large thicknesses of both layers, since there is a much longer optical path along which light can be affected by its properties.

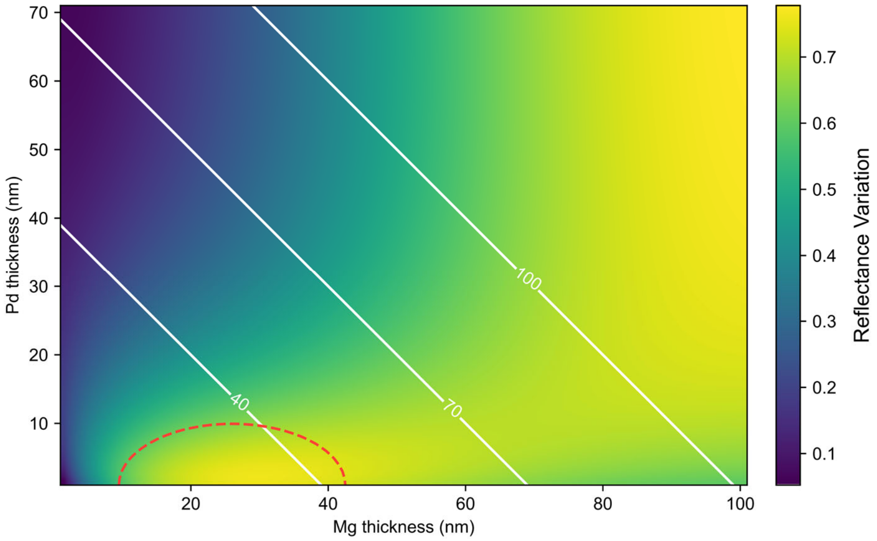

Figure S3. Calculated variation in reflectance upon hydrogenation at 1550 nm for a multi-layer structure with variable thicknesses of Mg and Pd. White contour lines indicate total structure thickness, while the red dashed line indicates boundaries of a region with high reflectance variation and low total thickness.

However, for non-extreme values of Mg thickness, some regions of the plot present a considerably larger variation than their surroundings. These are delimited by red dashed lines in Figure 3. While variations are very high for structures with total thickness over 100 nm (and specifically with over 80 nm of Mg), the issue of H_2 adsorption kinetics, and their impact on the sensor's response time, must be considered. This is because the quantities calculated here only relate to the initial and final states of hydrogenation, with no information or modelling of the phase transition dynamics.

Since one of the objectives of this work is to design a hydrogen sensor to quickly detect leaks, the focus of structure optimization should be placed on obtaining a response that, while easily detectable, is as fast as possible. It is safe to assume that a larger amount of material that hydrogen must diffuse through would be highly counterproductive for this goal.

Therefore, it is adequate to look for structures with lower total thicknesses. In Figure S3, a region of larger variation values appears around a Mg thickness of 25 nm, for very thin Pd layers. This is, essentially, a structure that consists of an optimally-sized Mg layer (thick enough to provide a large change in reflectance, but still able to be quickly hydrogenated) with a Pd cap functioning only as an adsorption catalyst. Thus, Mg and Pd thickness ranges of 20-30 nm and up to 10 nm, respectively, seem suitable. Reliable fabrication of very thin (a few nanometers) films is, however, not trivial. Without very accurate and carefully calibrated deposition systems, a conformal, smooth film under 5 nm is not always easily achieved through sputtering methods. Furthermore, validation techniques such as SEM may not provide sufficient resolution to observe these structures except on very high-end and expensive systems. Thus, it may be more reliable to work with a Pd layer that is slightly thicker than ideal, to help guarantee film homogeneity and quality.

EDS Film Characterization

Energy-dispersive X-ray spectroscopy (EDS) was used to obtain a qualitative analysis of the composition of the produced thin films. For this, a 20 nm Mg film capped with a 10 nm Pd films was deposited on a silicon substrate with RF magnetron sputtering, using the same procedure employed for all the sensor devices presented in the article.

The EDS analysis was carried out using a ThermoFisher Phenom Pharos G2 Desktop FEG-SEM, with spectral processing and element labelling handled by the provided software. Several measurements were taken on different points of the sample's surface, yielding similar results.

Figure S4 shows a spectrum of one such measurement, where there is a large dominating peak for silicon, accompanied by two smaller peaks identified as Mg and Pd. No significant number of other elements was identified. Below are the quantitative results and measurement details:

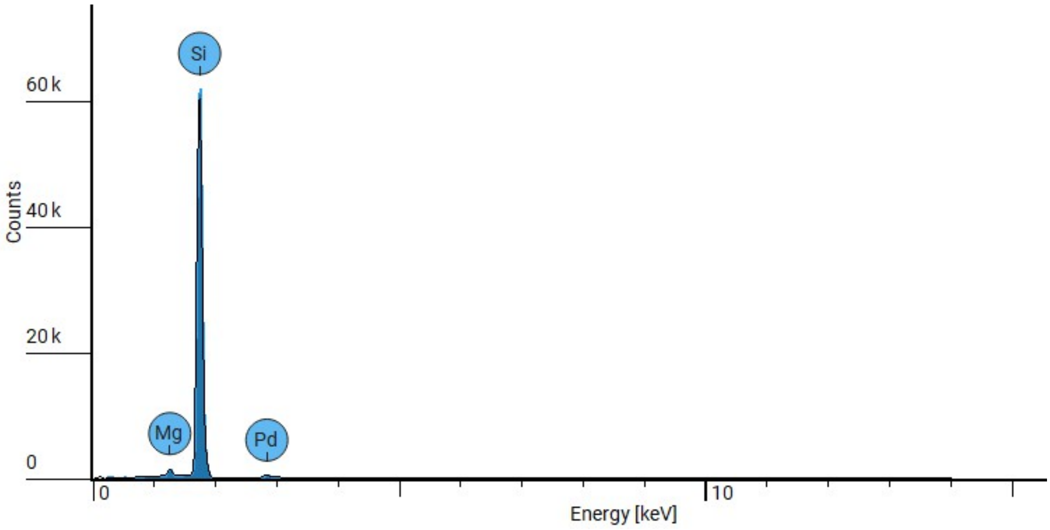

Figure S4. EDS spectrum for a 20 nm Mg film capped with 10 nm Pd, on a silicon substrate.

FW: 15 µm, Mode: 15 kV - Point, WD: 7.3 mm, Detector: BSD Full, Time: 4/16/26 2:38 PM

	Element Number	Element Symbol	Element Name	Atomic Conc [%]	Weight Conc. [%]
	12	Mg	Magnesium	1.43	1.20
	14	Si	Silicon	97.28	94.10
	46	Pd	Palladium	1.28	4.70

666 878 counts in 0:01:00 (11 103 c/s)

Disabled elements: -

Sensor Diagram and Photograph

The figures in this section illustrate and describe the structure and enclosure of the H2 sensing fiber tips. Figure S5 shows a 3D diagram of one such sensor, where the assembly of all the enclosure parts can be seen, along with a schematic of the thin film structure on the SM fiber tip (bottom insert). Figure S6 is a photograph showing the same components during the sensor enclosure assembly stage of fabrication.

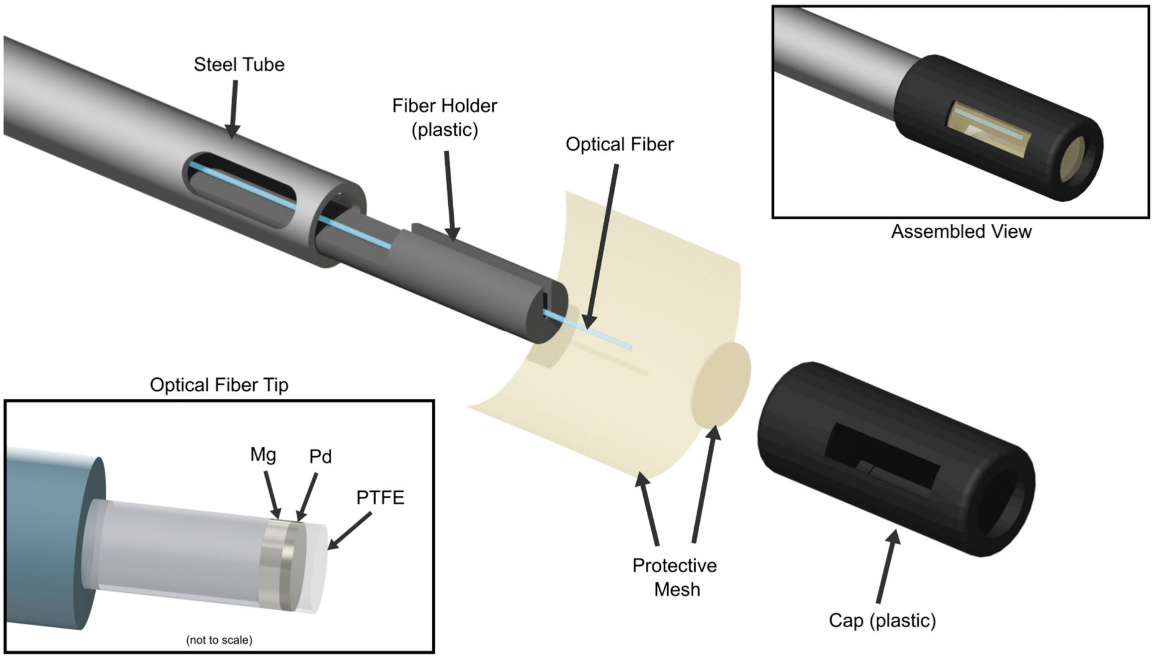

Figure S5. Diagram of the sensor, showing an exploded view of the optical fiber and its enclosure. The insets show an assembled view (top right) and a shematic of the optical fiber tip with the deposited thin films (bottom left).

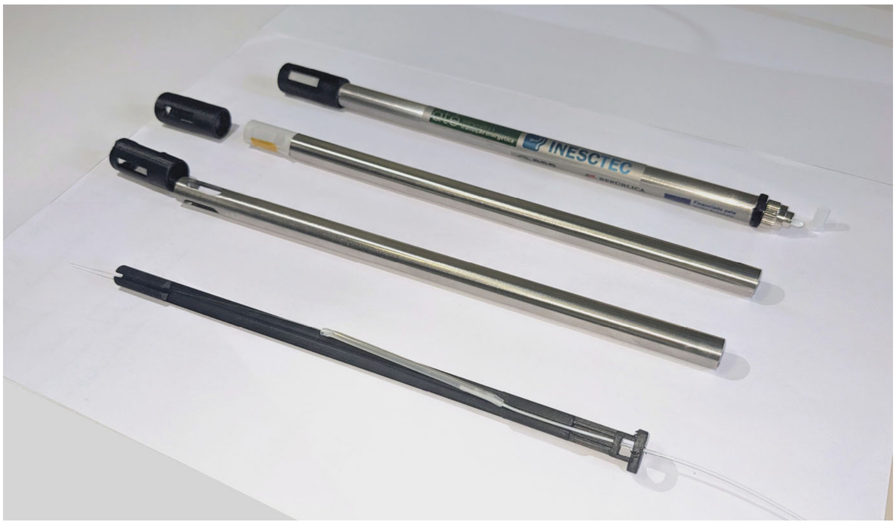

Figure S6. Photograph showing several sensor parts in different stages of enclosure assembly.
